# Point Prevalence Survey of Antimicrobial Use in a Malaysian Tertiary Care University Hospital

**DOI:** 10.3390/antibiotics10050531

**Published:** 2021-05-04

**Authors:** Nurul Adilla Hayat Jamaluddin, Petrick Periyasamy, Chee Lan Lau, Sasheela Ponnampalavanar, Pauline Siew Mei Lai, Ramliza Ramli, Toh Leong Tan, Najma Kori, Mei Kuen Yin, Nur Jannah Azman, Rodney James, Karin Thursky, Isa Naina-Mohamed

**Affiliations:** 1 Pharmacoepidemiology and Drug Safety Unit, Department of Pharmacology, Faculty of Medicine, Universiti Kebangsaan Malaysia, Cheras, Kuala Lumpur 56000, Malaysia; adilla@cyberjaya.edu.my; 2 Department of Hospital and Clinical Pharmacy, Faculty of Pharmacy, University of Cyberjaya, Cyber 11, Cyberjaya, Selangor 63000, Malaysia; 3 Medical Department, Faculty of Medicine, Universiti Kebangsaan Malaysia, Cheras, Kuala Lumpur 56000, Malaysia; petrick.periyasamy@gmail.com (P.P.); najmakori@ukm.edu.my (N.K.); 4 Pharmacy Department, Hospital Canselor Tuanku Muhriz, Cheras, Kuala Lumpur 56000, Malaysia; cllau@ppukm.ukm.edu.my (C.L.L.); mkyin@ppukm.ukm.edu.my (M.K.Y.); jannah@ppukm.ukm.edu.my (N.J.A.); 5 Department of Medicine, University of Malaya, Kuala Lumpur 50603, Malaysia; sheela@ummc.edu.my; 6 Department of Primary Care Medicine, University of Malaya, Kuala Lumpur 50603, Malaysia; plai@um.edu.my; 7 Department of Medical Microbiology and Immunology, Faculty of Medicine, Universiti Kebangsaan Malaysia, Cheras, Kuala Lumpur 56000, Malaysia; ramliza@ppukm.ukm.edu.my; 8 Department of Emergency Medicine, Faculty of Medicine, Universiti Kebangsaan Malaysia, Cheras, Kuala Lumpur 56000, Malaysia; sebastianttl@yahoo.co.uk; 9 National Centre for Antimicrobial Stewardship, Peter Doherty Research Institute for Infection and Immunity, Melbourne 3000, Australia; rodney.james@mh.org.au (R.J.); karin.thursky@mh.org.au (K.T.)

**Keywords:** antimicrobial, point prevalence, compliance, risk, prescribing

## Abstract

Antimicrobial resistance remains a significant public health issue, and to a greater extent, caused by the misuse of antimicrobials. Monitoring and benchmarking antimicrobial use is critical for the antimicrobial stewardship team to enhance prudent use of antimicrobial and curb antimicrobial resistance in healthcare settings. Employing a comprehensive and established tool, this study investigated the trends and compliance of antimicrobial prescribing in a tertiary care teaching hospital in Malaysia to identify potential target areas for quality improvement. A point prevalence survey method following the National Antimicrobial Prescribing Survey (NAPS) was used to collect detailed data on antimicrobial prescribing and assessed a set of quality indicators associated with antimicrobial use. The paper-based survey was conducted across 37 adult wards, which included all adult in-patients on the day of the survey to form the study population. Of 478 patients surveyed, 234 (49%) patients received at least one antimicrobial agent, with 357 antimicrobial prescriptions. The highest prevalence of antimicrobial use was within the ICU (80%). Agents used were mainly amoxicillin/β-lactamase inhibitor (14.8%), piperacillin/β-lactamase inhibitor (10.6%) and third-generation cephalosporin (ceftriaxone, 9.5%). Intravenous administration was ordered in 62.7% of prescriptions. Many antimicrobials were prescribed empirically (65.5%) and commonly prescribed for pneumonia (19.6%). The indications for antimicrobials were documented in the patients’ notes for 80% of the prescriptions; however, the rate of review/stop date recorded must be improved (33.3%). One-half of surgical antimicrobial prophylaxis was administered for more than 24 h. From 280 assessable prescriptions, 141 (50.4%) were compliant with guidelines. Treating specialties, administration route, class of antimicrobial, and the number of prescriptions per patient were contributing factors associated with compliance. On multivariate analysis, administering non-oral routes of antimicrobial administration, and single antimicrobial prescription prescribed per patient was independently associated with non-compliance. NAPS can produce robust baseline information and identifying targets for improvement in antimicrobial prescribing in reference to current AMS initiatives within the tertiary care teaching hospital. The findings underscore the necessity to expand the AMS efforts towards reinforcing compliance, documentation, improving surgical prophylaxis prescribing practices, and updating local antibiotic guidelines.

## 1. Introduction

Antimicrobials were once hailed as a ‘medical miracle’ [1] and have significantly impacted the prognosis of patients with severe infectious diseases over the past 60 years [2]. However, the growth and dissemination of resistant organisms have compromised the effectiveness of antimicrobials [2]. While the underlying reason behind this dynamic and mounting problem is undoubtedly the amount of antimicrobial use in general, massive consumption, abuse, and misuse of antimicrobials, which is influenced by several interrelated factors, have substantially contributed to speed up the spread of resistant pathogens [3,4]. World Health Organisation (WHO) advocates the adoption of antimicrobial stewardship (AMS) initiatives to monitor antimicrobial use and tackle the antimicrobial resistance (AMR) burden [5]. Additionally, the Centers for Disease Control and Prevention (CDC) recommends antibiotic usage surveillance as part of the AMS programme’s core elements [6]. With a tremendous rise in superbug resistance in Malaysian hospitals, an effective approach to the AMS programme is needed to improve antimicrobial use and curb AMR in Malaysian health care facilities [7]. Numerous prevalence studies on antimicrobial prescribing in hospital settings using established and standardised surveillance tools were previously reported, and most of these studies, however, were conducted in developed countries. An established and standard surveillance tool is essential for sustainable AMS efforts in audit training, data collection, classification, storage, retrieval, analysis, and presentation of large amounts of health data, facilitating data comparability and benchmarking over time while focusing on critical key indicators. It also complements the AMS initiatives to consistently monitor the quality of prescribing and the effect of interventions to promote judicious prescribing over time.

Hence, this study presents current antimicrobial prescribing prevalence in adult in-patients by evaluating the patterns and compliance of antimicrobial prescriptions with national antibiotic guidelines and local protocols at a Malaysian tertiary teaching hospital. Furthermore, we sought to recognise the possible factors associated with (non-) compliance to inform future AMS interventions. To our knowledge, this is the first prevalence report on hospital antimicrobial epidemiology adopting an established and standardised Hospital National Antimicrobial Prescribing Survey (NAPS) method, a key component of the Antimicrobial Use and Resistance in Australia (AURA) Surveillance System. NAPS was developed by National Centre for Antimicrobial Stewardship (NCAS) in collaboration with the Australian Commission on Safety and Quality in Health Care. The validated method was designed to assist healthcare facilities to assess the prevalence of antimicrobial use and the quality of antimicrobial prescribing. NAPS is also designed to monitor the performance of the AMS programme in an institution.

## 2. Results

### 2.1. Demographics and Prevalence

A total of 478 patients were identified through the hospital system database in 37 adult wards during the survey period, including ICU, burn unit, and a mixed ward. Overall, 234 (49%) patients received at least one antimicrobial agent, for a total of 357 antimicrobial prescriptions. Patients’ age ranged from 16–93 years, with a mean age of patients receiving antibiotic therapy was 59.96 (SD 17.25) years. The age group with the highest admissions during the survey was between 65- to 79-year-olds, which constituted 35.9% of all patients. Of these, 56.4% were male. Patients were classified according to treating speciality teams: medical, surgical, orthopaedic, obstetrics and gynaecology (O&G), intensive care, oncology; and the largest pool (52.1%) was from medical specialities’ wards. Seven (3%) patients were on dialysis. There were 53 different antimicrobials prescribed for 119 indications, with 69.2% of patients receiving a single antimicrobial, 19.2% receiving two antimicrobials, and 11.5% receiving three or more agents. Forty-two (18%) patients received at least one pathogen-directed antimicrobial prescription based on confirmed culture and antimicrobial sensitivity test (AST) results. The characteristics of patients are displayed in Table 1.

### 2.2. Prescription Rate and Antibiotic Use

**Figure 1:**
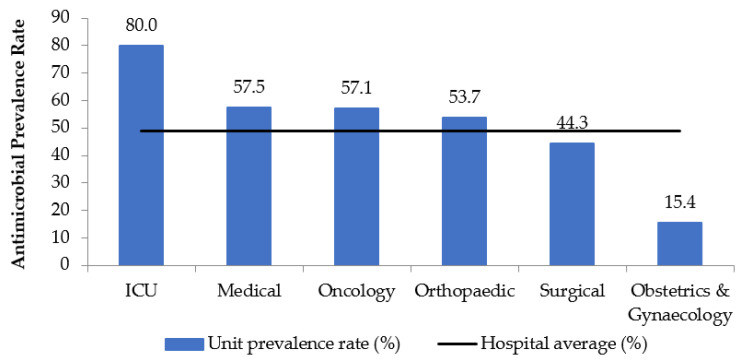
Antimicrobial prescription rates in various units.

The highest prescription rate of antimicrobials based on specialties was in ICU (80%), followed by medical (57.5%), oncology (57.1%), and orthopaedic (53.7%). The surgical (44.3%), and O&G (15.4%) had an antimicrobial prescription rate below the hospital average (Figure 1).

The top five indications for antimicrobials prescriptions were medical prophylaxis (41 prescriptions, 11.5%), followed by empiric therapy for community-acquired pneumonia (36 prescriptions, 10.1%), surgical prophylaxis (24 prescriptions, 6.7%), Mycobacterium tuberculosis (22 prescriptions, 6.2%), and empiric therapy of hospital-acquired pneumonia (22 prescriptions, 6.2%). These indications were more commonly recorded than a diagnosis of more severe infection, sepsis (19 prescriptions, 5.3%). Among the surgical prophylaxis orders, 12 (50%) had been prescribed for longer than 24-h post-surgery. In contrast to directed therapy to a known pathogen (16.2%), empirical therapy (65.5%) was reported with the highest prevalence in the medical, surgical, and orthopaedic units.

**Figure 2:**
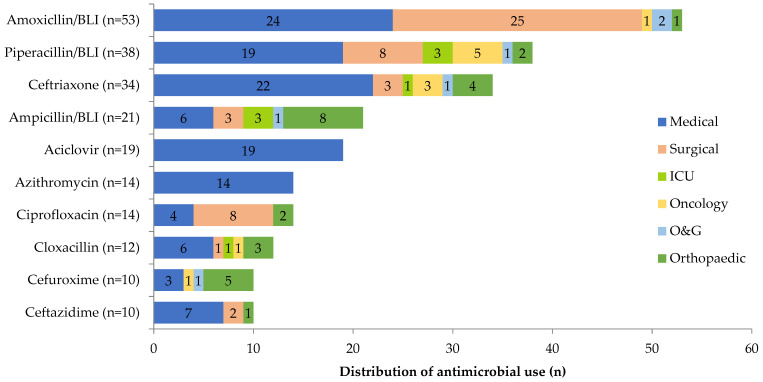
Distribution of 10 most prescribed antimicrobials in various units. The total number of prescriptions is displayed next to each antimicrobial name. BLI: β-lactamase inhibitor.

β-lactam with/without β-lactamase inhibitor was the most used antimicrobial class. The antimicrobial agents used were mainly amoxicillin/β-lactamase inhibitor (14.8%), piperacillin/β-lactamase inhibitor (10.6%), third-generation cephalosporin (ceftriaxone) (9.5%), ampicillin/β-lactamase inhibitor (5.9%) and acyclovir (5.3%). The amoxicillin/β-lactamase inhibitor was mostly prescribed for surgical (47.2%), while piperacillin/β-lactamase inhibitor and ceftriaxone usage were mainly reported among medical patients, 50% and 64.7%, respectively. The highest consumption of ampicillin/β-lactamase inhibitor was by the orthopaedic unit, whereas acyclovir was prescribed for medical prophylaxis purposes within the medical unit (Figure 2).

The antimicrobials were mainly administered via the parenteral route (62.7%), with the highest prevalence recorded in ICU (76.1%). The indication of antimicrobial was documented in most of the prescriptions (80.4%). However, the review or stop date plan of antimicrobial therapy was not properly documented in many cases (66.7%). Overall, assessment on 280 prescriptions (excluding prescriptions for which guidelines were not available, directed therapy, and not assessable) revealed that one-half (50.4%) were compliant with the primary recommendations by national antibiotic guidelines and local endorsed protocols (Table 2).

Of 58 pathogen-directed therapy prescriptions, amoxicillin/β-lactamase inhibitor (15.5%) was found the most prescribed antimicrobial mainly targeting *Klebsiella* spp., *Escherichia coli,* and group B beta-hemolytic *Streptococcus aureus* (GBS). In total, 17.2% of pathogen-directed therapy prescriptions were targeting *Staphylococcus aureus*, the most common microorganism isolated during the survey. Overall, 87.9% of pathogen-directed antimicrobials were prescribed according to the available AST results. The kappa statistics revealed almost perfect agreement (k = 0.816, *p* < 0.001) between prescribed pathogen-directed antimicrobials and AST.

### 2.3. Factors Influencing Compliance

In univariate analyses (per prescription), the following factors were associated with lower compliance with guidelines: treating specialities, administration route, class of antimicrobial, and the number of prescriptions per patient. The prescriptions from O&G (OR 13.70, 95% CI 1.70–110.53 *p* < 0.001), orthopaedic (OR 4.57, 95% CI 1.73–12.07 *p* < 0.001), oncology (OR 2.74, 95% CI 0.88–8.52 *p* < 0.001) and surgical unit (OR 2.25, 95% CI 1.19–4.23 *p* < 0.001) were associated with higher odds for non-compliant with guidelines compared to medical and ICU. Non-compliance was also more commonly identified in the consumption of the cephalosporin class of antimicrobials (OR 2.24, 95% CI 1.06–4.76, *p* < 0.001). Multivariate analysis revealed administration route and number of prescriptions per patient remained independently associated with an increased odds ratio for non-compliant with the primary recommendations. Parenteral antimicrobial (OR 2.22, 95% CI 0.93–5.31, *p* = 0.072) and other non-oral routes of administration (OR 19.05, 95% CI 4.19–86.60, *p* < 0.001) were associated with a higher likelihood of being non-compliant compared to prescribing oral antimicrobial. A larger number of prescription (≥ 3) prescribed per patient or combination therapy resulted in an increased compliant (OR 0.21, 95% CI 0.08–0.53, *p* < 0.001) in each prescription when measured against a smaller number of prescription or monotherapy (Table 3).

## 3. Discussion

This present study describes a unique antimicrobial prescribing data set from 234 adult patients across a teaching hospital using the NAPS data collection tool. The prevalence of antimicrobial use was 49%, which is in line with the recent prevalence reported for public hospitals across Malaysia, ranging from 32.7% to 60% [7]. It is of concern as it indicated a lack of improvement shown during the last decade in a crucial area in the fight against AMR. This was also comparable to the average prevalence observed among hospitals in the neighbouring country, Singapore (51%) [8]. Figures for the prevalence of antimicrobial consumption to adult hospitalised patients in other PPS studies varied between different regions; 43% in Australia [9], 56% in China [10], 32.9% in Europe [11], 38% in Canada [12], and 34.4% from the reported data collected across 53 countries [13].

The highest rate of antimicrobial usage in this study was found in the ICU, where 80% of patients were placed on antimicrobials therapy. Most cases had been admitted for more than seven days prior to the day of the survey, and the prescriptions in ICU were found to be mostly microbiologically confirmed treatment (71.4%). Likewise, as reported in other PPS studies among the adult population in Nigeria [14], Pakistan [15], and Brazil [16], greater prescribing of antimicrobial in ICU compared with other hospital in-patient admission sites was due to the critical state of health of patients in the unit, with more severe infections, who also had more co-existing medical conditions. Additionally, an increased frequency of hospital-acquired infections in low- and middle-income countries leads to prolonged and higher antimicrobial usage in the intensive care unit [17].

Compared to the Malaysian nationwide survey [7] trends in 2015–2016, empirical therapy (65.5%) was more commonly prescribed in this study, notably across medical (74.8%), surgical (58.6%), oncology (86.7%), and orthopaedic (55.6%) units. The reason is uncertain and probably multifactorial. Most cases receiving initial therapy were most likely based on clinical judgements and experience while awaiting laboratory results that were delayed by limited resources and technology advancement. Identification of the causative pathogens facilitated the correct diagnosis, de-escalation, and use of targeted agents, which are necessary to promote the prudent use of antimicrobials. Nevertheless, in clinical practice, it is not always possible to identify the aetiology, and the susceptible treatment is sometimes limited by the host factors.

Pneumonia (community-acquired, hospital-acquired, and aspiration) was by far the most common indication for antimicrobial treatment (19.6%), which was largely (94.3%) empirically treated. The respiratory infection was reported in a moderate proportion of hospitalised patients in Malaysia [18]. At the time of the survey, 18 patients received antimicrobial for surgical prophylaxis, and 24 prescriptions were issued. The extended duration of surgical antimicrobial prophylaxis >24 h was found in half (50%) of the surgical prophylaxis prescriptions, ranging from 33.3% in O&G to 85.7% in the orthopaedic unit, which was contrary to accepted evidence-based practices and target for this indicator of less than 5% [19]. The results were higher than the NAPS in Australian hospitals (30.5%) [19]. A similar situation or higher rates of surgical antimicrobial prophylaxis >24 h have been reported in other countries (range 52.8–77%) [11,12,20,21,22]. A survey conducted among general surgeons found that failure to keep up to date, reliance on habit rather than on evidence-based practices, personal experience and preference, peer influence, and institutional norms affected their choice of duration of antimicrobials cover [23]. Prolonging antimicrobial prophylaxis >24 h for most surgical procedures does not prevent the development of postoperative infections compared to recommended duration (24 h or less). On the other hand, induces AMR with adverse effects. In the absence of preoperative infection or severe complications, a prolonged postoperative antibiotic is unnecessary [24].

Penicillin with a β-lactamase inhibitor (amoxicillin/β-lactamase inhibitor, piperacillin/β-lactamase inhibitor, ampicillin/β-lactamase inhibitor) and third-generation cephalosporin (ceftriaxone) was prescribed in a substantial amount of prescriptions which corresponds with the utilisation trend in Malaysian hospitals from 2012–2016 [7] and other international reports [13,20,25]. Amoxicillin/β-lactamase inhibitor was prescribed commonly for community-acquired pneumonia (18.9%) and surgical prophylaxis (18.9%). Although it is available in an oral form, 83% of the prescriptions containing amoxicillin/β-lactamase inhibitor was ordered for an intravenous route. Piperacillin/β-lactamase inhibitor was prescribed mainly for empiric therapy of hospital-acquired pneumonia (26.3%) and sepsis (26.3%). Ampicillin/β-lactamase inhibitor was a preferred choice for the treatment of skin and soft tissue infections (66.7%). In this study, acyclovir was an antiviral used for medical prophylaxis among haematological patients receiving chemotherapy. The high proportion of cephalosporin usage in this present study is a concern. Ceftriaxone prescribed for surgical prophylaxis (20.6%) and empirical therapy of pneumonia (50%) deviated from the primary recommendation in the guidelines and hospital protocols.

Parenteral antimicrobials (67.2%) were observed in this study and are likely related to the high usage of agents mainly available in injection form, namely piperacillin/β-lactamase inhibitor, ceftriaxone, and ampicillin/β-lactamase inhibitor regardless of empiric or directed therapy. The use of parenteral therapy is inevitable in patients admitted with severe infections and often prompted by their life-threatening conditions, oral intolerance, age, type of lesion, microbial susceptibility, and availability of dosage form. Other similar studies have likewise identified a high rate of prescribing the parenteral antimicrobial among hospitalised patients [13,14,15,26,27]. In addition, physicians prefer to use the injection and generally believe in the superiority of the intravenous antimicrobial, although not always supported by good evidence [28,29]. For many indications and circumstances, opting for intravenous therapy may not be the most beneficial choice, and the risks (catheter-related complications, healthcare costs, length of hospital stay) are well established. Intravenous to oral antimicrobial conversion obviates these negative impacts and is recognised as a key parameter for stewardship processes in hospitals [13,26,30,31]. On another note, the present study showed the use of topical antimicrobials and intraperitoneal routes has a significantly higher non-compliant rate.

The documentation of the reason for prescribing and review or stop date of antimicrobial therapy is an important quality indicator because it ensures communication of diagnosis and subsequent therapy plan among treating teams and other healthcare providers. Despite manual medical recording and charting systems in the hospital, the documentation of indication rate by prescribers (80.4%) was comparable to previous reports (range 76.9% to 87.8%) [19,25,27]. However, it still requires improvement to meet the NAPS target and international standard of > 95% [20,32,33,34]. In cases where the indications were not recorded, the rationale for the antimicrobial prescription was missing, and this remains an ongoing challenge for further assessment. Moreover, the documentation rate of review or stop date plan was not optimal. Proper documentation should be encouraged as part of good antimicrobial prescribing practice to prevent unnecessary prolonged antimicrobial use. Although a low level of documentation of review and stop antimicrobial date was commonly evidenced in multicentre international surveys (range 27.8–56.3%) [13,19,21,25,35], NAPS regarded the level of review/stop date in this study as unsatisfactory, far behind the target of >95%.

An international antibiotic policy suggests that 90% of antimicrobial prescriptions should be in congruence with guidelines [25], highlighting the need to improve in this component. The overall rate of compliance was relatively low (50.4%) compared to published work in Australia (67.3%) [19] and other studies [21,25], with slightly better compliance in a medical unit (60.3%). In contrast, several studies marked the highest guideline compliant in ICU [21,36]. More often than not, prescribing outside guidelines is always linked to senior colleagues’ influence, clinical autonomy, and experience of the prescriber [37]. As for the present survey, the deviation explained the underutilisation of hospital protocol established based on local antibiogram, as well as lack of a local antibiotic guideline that tailors to the resistance and susceptibility profile, and adapted for local needs in this hospital. It is critical to enhancing compliance in prescribing by developing and updating comprehensive local evidence-based guidelines periodically, and an AMS programme that offers guidance to others. Our study signified better guideline compliance when multiple prescriptions were administered to a patient diagnosed with an infection or co-existing infections requiring and receiving combination therapy. The relatively low rate of non-compliance could be partly attributed to the complexity and severity of diseases, which require the active participation of specialists from infectious disease, microbiology, and pharmacists in these patients’ routine care [38]. The use of broad-spectrum cephalosporin, parenteral, and other than oral form antimicrobials were significantly associated with the low guideline compliance indicating additional measures, such as pre-authorisation, post-prescription review, or timely AST report and interpretation, are desirable.

## 4. Materials and Methods

### 4.1. Study Design and Settings

A cross-sectional audit of antimicrobial prescribing was performed at the Hospital Canselor Tuanku Muhriz (HCTM), a university-affiliated hospital with a 900-bed tertiary care centre in Kuala Lumpur, Malaysia.

The hospital-wide point prevalence survey (PPS), using the Hospital NAPS tool, was executed by 14 clinical pharmacists from 16 to 30 April 2019. All adult wards were audited once. Patients were identified through the Caring Hospital Enterprise System (CHEtS) database, which receives real-time admission/discharge and transfer inputs and reviewed in the ward. The paper-based survey included all in-patients who were on the wards at 0800 h on the survey day. Data collection was done with two forms: one for ward-level data to record the denominators, i.e., the total number of in-patients on the ward, and one for patient-level data to record numerators. All antimicrobials (antibiotics, antivirals, antifungals, antiparasitics) were recorded on the patient’s medical records (medication charts, surgical or procedural records) prescribed at 0800 a.m. on the audit morning, given via all formulations (systemic and topical) were noted. When multiple antimicrobials were prescribed to a patient, all were recorded.

For each patient with an antimicrobial prescribed and charted, the following information was obtained and entered on the data collection form: demographic, diagnosis, indications, dosage, route, frequency, and duration, which included start and review/stop date. If not documented, the indication for antimicrobial therapy was interpreted by the surveyor based on obvious information available in the medical records. Data was also collected for any patient who was prescribed a stat dose of an antimicrobial or surgical antibiotic prophylaxis since 0800 a.m. the previous day. The patient’s microbiology, haematology, and biochemistry laboratory data were retrieved from Order Management System (OMS). Outpatients, patients in daycare or emergency unit but not admitted into the wards yet, and patients in psychiatric wards were excluded. All study data were completely anonymised, and each patient record was given a unique, non-identifiable survey number.

Prior to the survey day, video and webinar training regarding the surveillance protocol were provided to all surveyors. Technical and clinical supports were available by email liaison with the NAPS personnel. Survey data were submitted online via a secure web-based platform. The quality indicators assessed included the following: guideline compliance, the reason for antimicrobial use in documentation, stop/review date documentation, and treatment type.

### 4.2. Definition

#### 4.2.1. Empiric/Directed/Prophylaxis

Antimicrobial therapy was identified as being empiric, directed, and prophylactic. Empiric treatment was defined as treatment that was started for a presumed possible infection without the infecting organism being identified. Directed therapy was a treatment ordered when pathogenic microorganism causing infection was identified upon the availability of microbiological culture or susceptibility results. Prophylaxis was defined as the use of an antimicrobial agent to prevent the patient from acquiring an infection and could either be surgical prophylaxis to prevent postoperative infections or medical prophylaxis to prevent infections among patients with immunodeficiency (examples pneumocystic pneumonia prophylaxis, preterm premature rupture of membranes prophylaxis).

#### 4.2.2. Compliance

The compliance of antimicrobial use was determined for each prescription as per the NAPS’s protocol assessment criteria based on primary references. The assessment was based on Malaysian national antibiotic guideline 2014 and standards for surgical prophylaxis were referred to an endorsed hospital surgical prophylaxis guide. A Guide to Antimicrobial Therapy in the Adult ICU was used to judge cases in the intensive care unit (ICU), and all doses were evaluated using endorsed hospital renal dose adjustment protocol. Guideline compliance is defined as prescribing the recommended first-line or preferred agent, route, dose, and frequency, according to the above guidelines and protocols, and evaluation was done based only on the information written in the patient records. In cases where recommendations in the primary references were found lacking or unclear, the assessment was based on a consensus of the two experts with/without referring to other supplemental references (i.e., international guidelines and ward protocols). Cases were recorded as compliant, non-compliant, non-assessable due to insufficient reports and unclear diagnosis, or no guidelines available.

### 4.3. Data Analysis

IBM SPSS statistics version 23.0 (IBM Corp., Armonk, NY, USA) was used for data analysis, descriptive analysis (percentage and frequency), categorical (mean ± SD), and continuous data variables. The study focused on prescribing antimicrobials during the survey period, which reported the number of treated patients and the number of prescriptions. A prescription was defined as the use of one agent by one route of administration. Antimicrobial prescribing rates were expressed as a percentage of patients receiving antimicrobial (proportional use). Univariate analysis using chi-square and Fisher’s exact tests for comparisons of categorical variables were employed where appropriate. A multivariate logistic regression model was performed to adjust the effects of potential factors that were significant in simple logistic regression (*p* < 0.05). The selection of variables was based on biological plausibility and demonstrated association in other previous literature [39]. The percentage of agreement and Kappa coefficient were used to investigate an agreement between pathogen-directed therapy prescribed and AST results.

### 4.4. Strengths and Limitations

The strengths of this study are inherent to the simplicity of the NAPS protocol, data collection tools, and templates, essential and needful support from the NAPS team. The study acknowledges the limitations of cross-sectional survey design, where only prevalence can be reported, and patients were not followed up in time. However, this design has been shown to provide reliable outcomes that can guide in identifying targets for intervention. In addition, the results were not corrected for several elements, namely patient case mix, disease incidence, institutional factors, or differences in climates and seasons, among other factors; all of which can influence antimicrobial use. As numerous surveyors were involved in the data collection, the assessments involved some degree of interpretation; there may be differences in the interpretations of data from incomplete charts or medical notes, potentially leading to discrepancies in assessments.

## 5. Conclusions

NAPS is reliable and capable of identifying priorities for antimicrobial prescribing quality improvement and establishing their baseline. A standard hospital-wide PPS using NAPS should be embedded, where possible, into routine AMS programmes for local and national levels for appropriate benchmarking. Several areas of practice deserve specific attention to optimise the prudent use of antimicrobial dispensing in this hospital. Future AMS initiatives should be directed towards unjustified deviation of therapy from the primary recommendation, particularly in the use of broad-spectrum, non-oral antimicrobials, and surgical prophylaxis prescribing practices. There is an opportunity to enhance quality in documenting indications and reporting a stop/review date.

## Figures and Tables

**Table 1:** Characteristics of patients receiving antimicrobial prescriptions (*n* = 234).

Characteristics	*n*	%
Age (year)		
Mean (SD)	59.96 (17.25)	
Age group		
16–29	15	6.4
30–49	43	18.4
50–64	66	28.2
65–79	84	35.9
≥80	26	11.1
Gender		
Male	132	56.4
Treating specialities		
Medical	122	52.1
Surgical	51	21.8
Orthopaedic	29	12.4
Obstetrics & Gynaecology	8	3.4
Intensive care unit	12	5.1
Oncology	12	5.1
Renal replacement therapy/dialysis		
Yes	7	3.0
Directed antimicrobial therapy		
Yes	42	18.0
Number of antimicrobial prescribed		
1	162	69.2
2	45	19.2
≥3	27	11.5

**Table 2:** Quality indicators for antimicrobial prescriptions (*n* = 357).

	Prescription by Specialities, *n* (%)					
Indicators (Number of Prescriptions, *n*)	Medical	Surgical	ICU	Oncology	O&G	Orthopaedic
	202 (56.5%)	70 (19.6%)	21 (5.9%)	15 (4.2%)	13 (3.6%)	36 (10.1%)
**Treatment**						
Empiric treatment (234)	151 (64.5)	41 (17.5)	6 (2.5)	13 (5.5%)	3 (1.3)	20 (8.5)
Directed therapy (58)	17 (29.0)	15 (26.0)	15 (26.0)	0	2 (3.4)	9 (15.5)
Prophylaxis (65)	34 (52.3)	14 (21.5)	0	2 (3.0)	8 (12.3)	7 (10.7)
Medical (41)	34 (82.9)	0	0	2 (4.8)	5 (12.2)	0
Surgical (24)	0	14 (58.3)	0	0	3 (12.5)	7 (29.1)
**Route of administration**						
Intravenous (224)	109 (48.7)	48 (21.4)	16 (7.1)	13 (5.8)	8 (3.6)	30 (13.4)
Oral (108)	82 (76.0)	11 (10.2)	5 (4.6)	2 (1.9)	4 (3.7)	4 (3.7)
Other (25)	11 (44.0)	11 (44.0)	0	0	1 (4.0)	2 (8.0)
**Reason for use documented**						
Yes (287)	172 (59.9)	42 (14.6)	20 (7.0)	14 (4.9)	8 (2.8)	31 (10.8)
No (70)	30 (42.9)	28 (40.0)	1 (1.4)	1 (1.4)	5 (7.1)	5 (7.1)
**Stop/review date documented**						
Yes (119)	82 (68.9)	8 (6.7)	10 (8.4)	1 (0.8)	9 (7.6)	9 (7.6)
No (238)	120 (50.4)	62 (26.0)	11 (4.6)	14 (5.9)	4 (1.7)	27 (11.3)
**Compliance with guideline**						
Yes (141)	105 (74.5)	21 (14.9)	3 (2.1)	5 (3.5)	1 (0.7)	6 (4.3)
No (139)	69 (49.6)	31 (22.3)	3 (2.2)	9 (6.5)	9 (6.5)	18 (12.9)
Not applicable (77) *	28 (36.4)	18 (23.4)	15 (19.5)	1 (1.2)	3 (3.9)	12 (15.6)

* Not applicable (directed therapy, no guidelines available for the specific indication, and not assessable compliance).

**Table 3:** Univariable and multivariable analysis of the associations between potential factors with guidelines noncompliance (*n* = 280).

	Compliant with Guideline		Univariate Analysis		Multivariate Analysis	
Factors	Compliant, Number of Prescriptions (%)	Non-Compliant, Number of Prescriptions (%)	Crude Odd Ratio for Non-Compliant (95% Confidence Interval)	*p*-Value	Adjusted Odd Ratio for Non-Compliant (95% Confidence Interval)	*p*-Value
**Specialities**						**0.231**
Medical	105 (60.3)	69 (39.7)	1.00 (Reference)	<0.001 ^b^	1.00 (Reference)	
Surgical	21 (40.4)	31 (59.6)	2.25 (1.19–4.23)		1.18 (0.57–2.46)	0.661
ICU	3 (50.0)	3 (50.0)	1.52 (0.30–7.76)		0.85 (0.15–4.77)	0.856
Oncology	5 (35.7)	9 (64.3)	2.74 (0.88–8.52)		1.72 (0.51–5.75)	0.379
O&G	1 (10.0)	9 (90.0)	13.70 (1.70–110.53)		9.54 (1.10–82.89)	0.041
Orthopaedic	6 (25.0)	18 (75.0)	4.57 (1.73–12.07)		2.26 (0.80–6.39)	0.123
**Route**						**0.001**
Oral	73 (80.2)	18 (19.8)	1.00 (Reference)	<0.001 ^a^	1.00 (Reference)	
Intravenous	64 (37.2)	108 (62.8)	6.84 (3.75–12.49)		2.22 (0.93–5.31)	0.072
Other *	4 (23.5)	13 (76.5)	13.18 (3.84–45.26)		19.05 (4.19–86.60)	<0.001
**Antimicrobial class**						**0.149**
Penicillin	41 (39.0)	64 (61.0)	1.00 (Reference)	<0.001 ^b^	1.00 (Reference)	
Cephalosporin	12 (22.2)	42 (77.8)	2.24 (1.06–4.76)		2.18 (0.94–5.06)	0.071
Quinolone	7 (50.0)	7 (50.0)	0.64 (0.21–1.96)		1.42 (0.28–7.620	0.673
Other **	29 (59.2)	20 (40.8)	0.44 (0.22–0.88)		0.63 (0.25–1.60)	0.334
Antifungal	13 (68.4)	6 (31.6)	0.30 (0.10–0.84)		1.05 (0.25–4.37)	0.947
Antiviral ^	25 (100.0)	0 (0.0)	0		NA	NA
Antituberculosis ^	14 (100.0)	0 (0.0)	0		NA	NA
**Number of prescriptions per patient**						**0.001**
1	49 (39.5)	75 (60.5)	1.00 (Reference)	<0.001 ^a^	1.00 (Reference)	
2	33 (45.2)	40 (54.8)	0.79 (0.44–1.42)		0.99 (0.44–2.24)	0.975
≥3	59 (71.1)	24 (28.9)	0.27 (0.15–0.48)		0.21 (0.08–0.53)	0.001

^a^ Chi-Squared test; ^b^ Fisher-Exact test, * Other routes include topical, intraperitoneal. Each of these route types accounted for <5% of prescriptions. ** Other classes include macrolide, carbapenem, imidazole. Each comprising < 5% of prescriptions. ^ Disregard due to the small number of cases.

## Data Availability

All data generated and analysed during this study have been included in this article.

## References

[1] Centers for Disease Control and Prevention (CDC) (1999). Achievements in Public Health, 1900–1999: Control of Infectious Diseases. MMWR Morb. Mortal. Wkly. Rep..

[2] Hulscher M.E., Grol R.P., van der Meer J.W. (2010). Antibiotic prescribing in hospitals: A social and behavioural scientific approach. Lancet Infect. Dis..

[3] Machowska A., Stålsby Lundborg C. (2018). Drivers of Irrational Use of Antibiotics in Europe. Int. J. Environ. Res. Public Health.

[4] Roca I., Akova M., Baquero F., Carlet J., Cavaleri M., Coenen S., Cohen J., Findlay D., Gyssens I., Heure O.E. (2015). The global threat of antimicrobial resistance: Science for intervention. New Microbes New Infect..

[5] World Health Organization (2015). Global Action Plan on Antimicrobial Resistance. http://apps.who.int/iris/handle/10665/193736.

[6] Centers for Disease Control and Prevention (2014). Core Elements of Hospital Antibiotic Stewardship Programs. http://www.cdc.gov/getsmart/healthcare/implementation/core-elements.html.

[7] Ministry of Health Malaysia (2017). Malaysian Action Plan on Antimicrobial Resistance (MyAP-AMR) 2017–2021. Minist. Heal. Malaysia.

[8] Cai Y., Venkatachalam I., Tee N.W., Yen Tan T., Kurup A., Wong S.Y., Low C.Y., Wang Y., Lee W., Liew Y.X. (2017). Prevalence of healthcare-associated infections and antimicrobial use among adult inpatients in Singapore acute-care hospitals: Results from the first national point prevalence survey. Clin. Infect. Dis..

[9] Ingram P.R., Seet J.M., Budgeon C.A., Murray R. (2012). Point-prevalence study of inappropriate antibiotic use at a tertiary Australian hospital. Intern. Med. J..

[10] Xie D., Xiang L., Li R., Hu Q., Luo Q., Xiong W. (2015). A multicenter point-prevalence survey of antibiotic use in 13 Chinese hospitals. J. Infect. Public Health.

[11] Plachouras D., Kärki T., Hansen S., Hopkins S., Lyytikäinen O., Moro M.L., Reilly J., Zarb P., Zingg W., Kinross P. (2018). Antimicrobial use in european acute care hospitals: Results from the second point prevalence survey (PPS) of healthcare-associated infections and antimicrobial use, 2016 to 2017. Eurosurveillance.

[12] Leung V., Li M., Wu J.H.C., Langford B., Zvonar R., Powis J., Longpre J., Béïque L., Gill S., Ho G. (2018). Evaluating antimicrobial use and spectrum of activity in Ontario hospitals: Feasibility of a multicentered point prevalence study. Open Forum Infect. Dis..

[13] Versporten A., Zarb P., Caniaux I., Gros M.F., Drapier N., Miller M., Jarlier V., Nathwani D., Goossens H., Koraqi A. (2018). Antimicrobial consumption and resistance in adult hospital inpatients in 53 countries: Results of an internet-based global point prevalence survey. Lancet Glob. Health.

[14] Oduyebo O., Olayinka A., Iregbu K., Versporten A., Goossens H., Nwajiobi-Princewill P., Jimoh O., Ige T., Aigbe A., Ola-Bello O. (2017). A point prevalence survey of antimicrobial prescribing in four Nigerian Tertiary Hospitals. Ann. Trop. Pathol..

[15] Saleem Z., Hassali M.A., Versporten A., Godman B., Hashmi F.K., Goossens H., Saleem F. (2019). A multicenter point prevalence survey of antibiotic use in Punjab, Pakistan: Findings and implications. Expert Rev. Anti. Infect. Ther..

[16] Porto A.P.M., Goossens H., Versporten A., Costa S.F. (2020). Global point prevalence survey of antimicrobial consumption in Brazilian hospitals. J. Hosp. Infect..

[17] World Health Organization Health Care-associated Infections. Fact Sheet. http://www.who.int/gpsc/country_work/gpsc_ccisc_fact_sheet_en.pdf.

[18] Azmi S., Aljunid S.M., Maimaiti N., Ali A.A., Muhammad Nur A., De Rosas-Valera M., Encluna J., Mohamed R., Wibowo B., Komaryani K. (2016). Assessing the burden of pneumonia using administrative data from Malaysia, Indonesia, and the Philippines. Int. J. Infect. Dis..

[19] National Centre for Antimicrobial Stewardship and Australian Commission on Safety and Quality in Health Care (2018). Antimicrobial Prescribing Practice in Australian Hospitals: Results of the 2017 Hospital National Antimicrobial Prescribing Survey.

[20] Ansari F., Erntell M., Goossens H., Davey P., Ii E., Care H., Group S. (2009). The European Surveillance of Antimicrobial Consumption ( ESAC ) Point-Prevalence Survey of Antibacterial Use in 20 European Hospitals in 2006. Clin. Infect. Dis..

[21] Singh S.K., Sengupta S., Antony R., Bhattacharya S., Mukhopadhyay C., Ramasubramanian V., Sharma A., Sahu S., Nirkhiwale S., Gupta S. (2019). Variations in antibiotic use across India: Multi-centre study through Global Point Prevalence survey. J. Hosp. Infect..

[22] Metsini A., Vazquez M., Sommerstein R., Marschall J., Voide C., Troillet N., Gardiol C., Pittet D., Zingg W. (2018). The Swissnoso Network Point prevalence of healthcare-associated infections and antibiotic use in three large Swiss acute-care hospitals. Swiss Med. Wkly..

[23] Gul Y.A., Lian L.H., Jabar F.M., Moissinac K. (2002). Antibiotic prophylaxis in elective colorectal surgery. ANZ J. Surg..

[24] Allegranzi B., Bischoff P., de Jonge S., Kubilay N.Z., Zayed B., Gomes S.M., Abbas M., Atema J.J., Gans S., van Rijen M. (2016). New WHO recommendations on preoperative measures for surgical site infection prevention: An evidence-based global perspective. Lancet Infect. Dis..

[25] Vandael E., Latour K., Goossens H., Magerman K., Drapier N., Catry B., Versporten A. (2020). Point prevalence survey of antimicrobial use and healthcare-associated infections in Belgian acute care hospitals: Results of the Global-PPS and ECDC-PPS 2017. Antimicrob. Resist. Infect. Control.

[26] Umeokonkwo C.D., Madubueze U.C., Onah C.K., Okedo-Alex I.N., Adeke A.S., Versporten A., Goossens H., Igwe-Okomiso D., Okeke K., Azuogu B.N. (2019). Point prevalence survey of antimicrobial prescription in a tertiary hospital in South East Nigeria: A call for improved antibiotic stewardship. J. Glob. Antimicrob. Resist..

[27] van Spreuwel P.C.J.M., Blok H., Langelaar M., Kullberg B.J., Mouton J.W., Natsch S. (2015). Identifying targets for quality improvement in hospital antibiotic prescribing. Neth. J. Med..

[28] Li H.K., Agweyu A., English M., Bejon P. (2015). An Unsupported Preference for Intravenous Antibiotics. PLoS Med..

[29] Broom J., Broom A., Adams K., Plage S. (2016). What prevents the intravenous to oral antibiotic switch? A qualitative study of hospital doctors’ accounts of what influences their clinical practice. J. Antimicrob. Chemother..

[30] Pollack L.A., Plachouras D., Sinkowitz-Cochran R., Gruhler H., Monnet D.L., Weber J.T. (2016). A concise set of structure and process indicators to assess and compare antimicrobial stewardship programs among EU and US Hospitals: Results from a multinational expert panel. Infect. Control Hosp. Epidemiol..

[31] Shrayteh Z.M., Rahal M.K., Malaeb D.N. (2014). Practice of switch from intravenous to oral antibiotics. Springerplus.

[32] Zarb P., Goossens H. (2011). European Surveillance of Antimicrobial Consumption (ESAC). Drugs.

[33] Health Protection Scotland (2011). Information Services Division. Scottish Antimicrobial Prescribing Group (SAPG) Report on Antimicrobial Use and Resistance in Humans in 2009. http://www.scottishmedicines.org.uk/SAPG/Scottish_Prescribing_Group__SAPG_.

[34] James R., Upjohn L., Cotta M., Luu S., Marshall C., Buising K., Thursky K. (2014). Measuring antimicrobial prescribing quality in Australian hospitals: Development and evaluation of a national antimicrobial prescribing survey tool. J. Antimicrob. Chemother..

[35] Al Matar M., Enani M., Binsaleh G., Roushdy H., Alokaili D., Al Bannai A., Khidir Y., Al-Abdely H. (2019). Point prevalence survey of antibiotic use in 26 Saudi hospitals in 2016. J. Infect. Public Health.

[36] Zarb P., Amadeo B., Muller A., Drapier N., Vankerckhoven V., Davey P., Goossens H., Metz-Gercek S., Jansens H., Markova B. (2011). Identification of targets for quality improvement in antimicrobial prescribing: The web-based ESAC point prevalence survey 2009. J. Antimicrob. Chemother..

[37] Skodvin B., Aase K., Charani E., Holmes A., Smith I. (2015). An antimicrobial stewardship program initiative: A qualitative study on prescribing practices among hospital doctors. Antimicrob. Resist. Infect. Control.

[38] Pulcini C., Botelho-Nevers E., Dyar O.J., Harbarth S. (2014). The impact of infectious disease specialists on antibiotic prescribing in hospitals. Clin. Microbiol. Infect..

[39] Grtler N., Erba A., Giehl C., Tschudin-Sutter S., Bassetti S., Osthoff M. (2019). Appropriateness of antimicrobial prescribing in a Swiss tertiary care hospital: A repeated point prevalence survey. Swiss Med. Wkly..

